# Anaerobic hydrocarbon degradation in candidate phylum ‘Atribacteria’ (JS1) inferred from genomics

**DOI:** 10.1038/s41396-019-0448-2

**Published:** 2019-06-06

**Authors:** Yi-Fan Liu, Zhen-Zhen Qi, Li-Bin Shou, Jin-Feng Liu, Shi-Zhong Yang, Ji-Dong Gu, Bo-Zhong Mu

**Affiliations:** 1 State Key Laboratory of Bioreactor Engineering and School of Chemistry and Molecular Engineering, East China University of Science and Technology, 200237 Shanghai, P.R. China; 2 School of Biological Sciences, The University of Hong Kong, Pokfulam Road, Hong Kong, P.R. China; 3 Shanghai Collaborative Innovation Center for Biomanufacturing Technology, 200237 Shanghai, P.R. China

**Keywords:** Bacterial genomics, Microbial ecology, Bacterial genomics, Microbial ecology, Bacterial genomics, Microbial ecology

## Abstract

The hydrocarbon-enriched environments, such as oil reservoirs and oil sands tailings ponds, contain a broad diversity of uncultured microorganisms. Despite being one of the few prokaryotic lineages that is consistently detected in both production water from oil reservoirs and stable hydrocarbon-degrading enrichment cultures originated from oil reservoirs, the physiological and ecological roles of candidate phylum “Atribacteria” (OP9/JS1) are not known in deep subsurface environments. Here, we report the expanded metabolic capabilities of Atribacteria as inferred from genomic reconstructions. Seventeen newly assembled medium-to-high-quality metagenomic assembly genomes (MAGs) were obtained either from co-assembly of two metagenomes from an Alaska North Slope oil reservoir or from previous studies of metagenomes coming from different environments. These MAGs comprise three currently known genus-level lineages and four novel genus-level groups of OP9 and JS1, which expands the genomic coverage of the major lineages within the candidate phylum Atribacteria. Genes involved in anaerobic hydrocarbon degradation were found in seven MAGs associated with hydrocarbon-enriched environments, and suggest that some Atribacteria could ferment short-chain *n*-alkanes into fatty acid while conserving energy. This study expands predicted metabolic capabilities of Atribacteria (JS1) and suggests that they are mediating a key role in subsurface carbon cycling.

## Introduction

Atribacteria is a candidate phylum that has been recently proposed to be inclusive of JS1 and OP9 lineages [1, 2]. Members from JS1 and OP9 are widely distributed in both the terrestrial and marine subsurface, including geothermal springs [3], anaerobic marine sediments [4, 5], petroleum reservoirs [6, 7], and brackish waters [8]. However, the understanding of the physiology and ecological role of these lineages has been hampered for decades owing to the lack of axenic cultures [9].

Recent studies based on culture-independent genomics have attempted to answer this question. With combined effort of single-cell sequencing and metagenomic binning, two genomes of related OP9-1 species, namely “*Ca*. Caldatribacterium californiense” and “*Ca*. Caldatribacterium saccharofermentans”, were retrieved from hot spring sediments in Little Hot Creek, USA and an in situ-enriched thermophilic cellulosic consortium (77CS) in Great Boiling Spring, USA, respectively [10]. Metabolic reconstruction suggested an anaerobic lifestyle for members of the OP9 lineage based on (hemi)cellulose fermentation through the Embden-Meyerhof glycolysis pathway with hydrogen, acetate and ethanol production in thermal environments. Later, Nobu et al. collected several previously available and heretofore unpublished OP9/JS1 SAGs from a terephthalate (TA)-degrading bioreactor, brackish waters of Sakinaw Lake, Canada, sediments of Etoliko Lagoon, Greece [8], and sediments of Aarhus Bay, Denmark [11]. In contrast to the OP9 lineage represented by “*Ca*. Caldatribacterium” spp., Atribacteria members from the JS1-1 and JS1-2 lineages were predicted to lack such sugar fermentation pathways but appear to have the capacity to catabolize organic acids such as propionate, which could be coupled with ethanologenic or acetogenic fermentation and syntrophic metabolism that depend on formate or H_2_ transfer [12, 13]. However, the existing genomic coverage only unveiled the tip of the iceberg of the diversity encompassed by this candidate phylum, especially when considering that as many as thirty-five genera within the phylum Atribacteria have been proposed, while only six genus-level lineages have been explored with genomic representatives, so far [1, 2].

Members of Atribacteria (OP9/JS1) are globally distributed, and in some cases abundant, in hydrocarbon-enriched environments, such as production water of oil reservoirs [7, 14, 15], methane hydrates [16, 17], marine hydrocarbon seeps [18] and stable anaerobic hydrocarbon-degrading cultures enriched from oil sands tailing ponds [19, 20], and hence an alternative role in hydrocarbon-enriched environments other than secondary fermenter was hypothesized for these microorganisms [20]. This hypothesis has recently been supported by results of the Schrader Bluff (SB) formation of Alaska, a highly degraded petroleum reservoir where Atribacteria were identified to be relatively abundant [21, 22]. Several of the recovered Atribacteria MAGs collected from this formation contained sequence fragments orthologous to alpha-subunits and gamma-subunits of benzylsuccinate synthase (*bssA* and *bssC*) [22], which have never been reported for atribacterial genomes obtained from other environments. Products encoded by *bssA*, together with its homologues *assA*/*masD*, *nmsA*, and *hbsA* are known to be fumarate addition enzymes (FAE) catalyzing the initial reaction of anaerobic hydrocarbon (alkyl-substituted benzene, *n*-alkane, methylnaphthalene, and *p*-cresol, respectively) degradation [23, 24]. However, further analysis of these putative *bss*-like genes was not undertaken and comprehensive phylogenetic and metabolic analysis of these OP9-like MAGs was hampered, probably owing to the small size (ranging from 0.2 to 0.9 MB) of recovered MAGs and hence low genome completeness (<23%, estimated by CheckM [25]) [22]. With increasing sequencing efforts, metagenomes derived from hydrocarbon-enriched environments have become available, and study of atribacterial MAGs obtained from these metagenomes might offer opportunity to reveal the phylogeny and physiology of FAE-containing members from this candidate phylum.

In this study, metagenome datasets from the Alaska North Slope, Schrader Bluff oil reservoir were downloaded and re-assembled using a co-assembly method. The high-quality (meet the standard proposed by Bowers et al. [26]) co-assembled Atribacteria-like MAGs, together with several other newly assembled medium-to-high-quality Atribacteria-like MAGs obtained from other studies were then analyzed for genes involved in anaerobic hydrocarbon degradation (fumarate addition and downstream pathways) and other potential metabolic characteristics of hitherto poorly-characterized lineages.

## Materials and methods

### Data availability

Except for the single cell amplified genomes (SAGs) and MAG described in previous studies [2, 10], MAGs (with contamination <8%, estimated by checkM [25]) which have been recently assembled from different metagenomes were download from National Center for Biotechnology Information (NCBI) database prior to May 12, 2018. Five of these MAGs were sourced from recent revaluation of publicly available metagenomes by Parks et al. [27]. These five genomes originated from hydrocarbon-enriched environments including Medicine Hat Glauconitic C (MHGC) oil reservoir, Canada (UBA6794 and UBA6251) [28], short-chain *n*-alkane (C6-C10) degrading culture SCADC (UBA4082) and naphtha-degrading culture NAPDC (UBA5772) which were collected from Mildred Lake Settling Basin (MLSB) tailings, Canada [29, 30], as well as metagenomes from a biogas reactors (UBA3950) [31]. Other atribacterial MAGs obtained from metagenomes of Crystal Geyser aquifer groundwater, USA (MAG-34_13, MAG-34_18, MAG-33_29, MAG-CG17, MAG-33_16, and MAG-33_13) [32], Rifle aquifer sediments, USA (MAG-RS-JS1, MAG-35_8, and MAG-35_14) [33] and Guaymas Basin sediments, USA (MAG-4572_76) [34] were also included in this study. Complete information on these MAGs is available in Supplementary Table S1. Additionally, two metagenomic datasets of Schrader Bluff Formation samples (SB1 and SB2) from Alaska North Slope oil reservoir were downloaded (accession number SRR1976948 and SRR1976948) for re-assembly (see below).

### Co-assembly and binning

The co-assembly of SB1 and SB2 metagenomes was performed as described before [35]. Briefly, metagenome datasets of Schrader Bluff Formation samples were downloaded and raw reads were quality-trimmed using Trim Galore! (http://www.bioinformatics.babraham.ac.uk/projects/trim_galore/) with default settings. Individual datasets were co-assembled using SPAdes v3.9.0 with the “meta” option [36]. Scaffolds were organized into genome bins based on tetranucleotide frequency and sequence coverage using Maxbin v2.2.3 with a marker genes set of 107 (Supplementary Table S2) [37]. Completeness and contamination levels of MAGs were estimated based on the representation of lineage specific tRNAs and marker genes sets (Supplementary Table S3) using CheckM v1.0.5 [25]. MAGs were de-contaminated using the RefineM v0.0.15 “outliers” commands which remove contigs from bins which appear to be outliers according to reference GC and tetranucleotide distributions in order to reduce contamination [25]. Further manual refinements to the MAGs were executed in Anvi’o using differential coverage, tetranucleotide frequency and marker gene content [38]. The Atribacteria-like MAGs in the co-assembled genome bins were identified if the open reading frames on the contigs had BLASTP hits >98% identity and covered >80% of the length of the contigs in the draft MAGs obtained from a previous study [22].

### Phylogenomic and phylogenetic analysis

Phylogenetic assignment of Atribacteria-like genomes was performed by retrieving 400 conserved marker genes (Supplementary Table S4) from genomes which were aligned using PhyloPhlAn, a de novo phylogenetic tree was then created using the “user” functionality based on a local database containing amino acid sequence files of Atribacteria-like genomes and reference genomes [39]. The newick format tree file was viewed and annotated using the online tool iTOL [40].

To calculate average amino acid identity (AAI) of shared orthologs between two Atribacteria-like genomes, a bidirectional top-scoring BLASTP approach (*E*-value <10^−5^) was utilized [41]. In brief, each amino acid sequence from a query genome was compared with a local database constructed from amino acid sequences file of another genome, using BLASTP. Then, the top hit was used as a query and compared with the original genome, using BLASTP. Pairwise orthologs was considered if the second BLASTP returns the first query sequence as top hit from the original genome.

To analyze phylogeny of individual genes in atribacterial FAE operons, amino acid sequences of each gene (*bssABCDEFG*, *assABCDEF*, *hbsABCDEF*, and *nmsABCDEF*) were retrieved individually from genomes of anaerobic hydrocarbon degradation microorganisms as reference sequences. Amino acid sequences files was passed to MAFFT and aligned using iterative progress methods “G-INS-i” [42]. Alignments were then checked and columns containing >95% gaps were trimmed in aliview [43]. Maximum-likelihood trees were constructed in the IQTREE Web Server with “standard” model and 1000 bootstrap alignments and viewed in MEGA7 [44, 45].

### Annotation and Identification of genes of interest

Protein coding genes and their associated translations in co-assembled MAGs and downloaded atribacterial MAGs were predicted using Prodigal v2.6 with “meta” option [46]. These amino acid sequences were then annotated for function using subsystem-based technology on the RAST server and using BlastKOALA to look for KEGG orthologues [47, 48]. Carbohydrate active enzymes (CAZY) were identified with dbCAN [49] using HMMER (v3) search [50] with an *E*-value threshold of 1E–18 and a coverage fraction cut-off of 0.35. For screening genes involved in specific pathways, such as anaerobic hydrocarbon degradation and fatty acid degradation, amino acid sequences were downloaded from the KEGG database and queried against the MAGs amino acid sequences using BLASTP. The identity of genes described in this study was further checked by BLASTP against the NCBI-nr database, and only the genes with top hits to relevant genes were kept.

Two standards were utilized to infer that a pathway was present. In the case of pathways that had already been identified in Atribacteria lineages (for example glycolysis in OP9-1 lineage), pathway were considered as present when 75% of the genes involved in the pathway were detected. As for pathways that haven’t been found previously in a given lineage (such as fatty acid degradation in OP9 and JS1 lineages), detection of genes involved in every step of the pathway was required.

## Results

To maximize assembly and get more complete MAGs, reads from the SB1 dataset and SB2 dataset were co-assembled and tools which are different from those used in the previous study for assembly and differential binning were used [22]. In total, four high-quality Atribacteria-like MAGs, Maxbin010, Maxbin015, Maxbin017, and Maxbin027, were retrieved from the co-assembly. The quality of Maxbin015 and Maxbin017 was much improved in terms of genome size, completeness and contamination compared with the previous corresponding draft MAGs of OP9_34_37 and OP9_34_686, respectively (Table 1) [22].

**Table 1:** Genome statistics of FAE-containing atribacterial MAGs

Genome	UBA4082	UBA5772	UBA6794	UBA6251	Maxbin015	Maxbin017	Maxbin027
Size (MB)	1.7	1.5	2	1.7	2.5/0.9	2.2/0.2	2.8
Completeness (%)	98.31	94.54	78.81	64.41	98.31/15.77	98.31/19.36	98.31
Contamination (%)	4.24	4.49	0	0	0/0	0/0	4.24
GC (%)	37.4	37.8	35.3	33.5	34.1/34.4	35.1/34.6	34.2
No. of scaffolds	78	129	85	67	107/155	87/56	424
N50 (bp)	31247	12962	36373	38862	106084/6790	66371/3664	30881
Genome quality	High	High	Medium	Medium	High/Low	High/Low	High
Resource	[27]	[27]	[27]	[27]	This study/[22]	This study/[22]	This study

The standard for high-quality, Medium-quality was suggested in [26]. The statistics of original draft MAGs of Maxbin015, Maxbin017 and Maxbin027 in previous study has been put after the slash for comparison [22]

### Phylogenetic analysis of Atribacteria-like MAGs

**Fig. 1:**
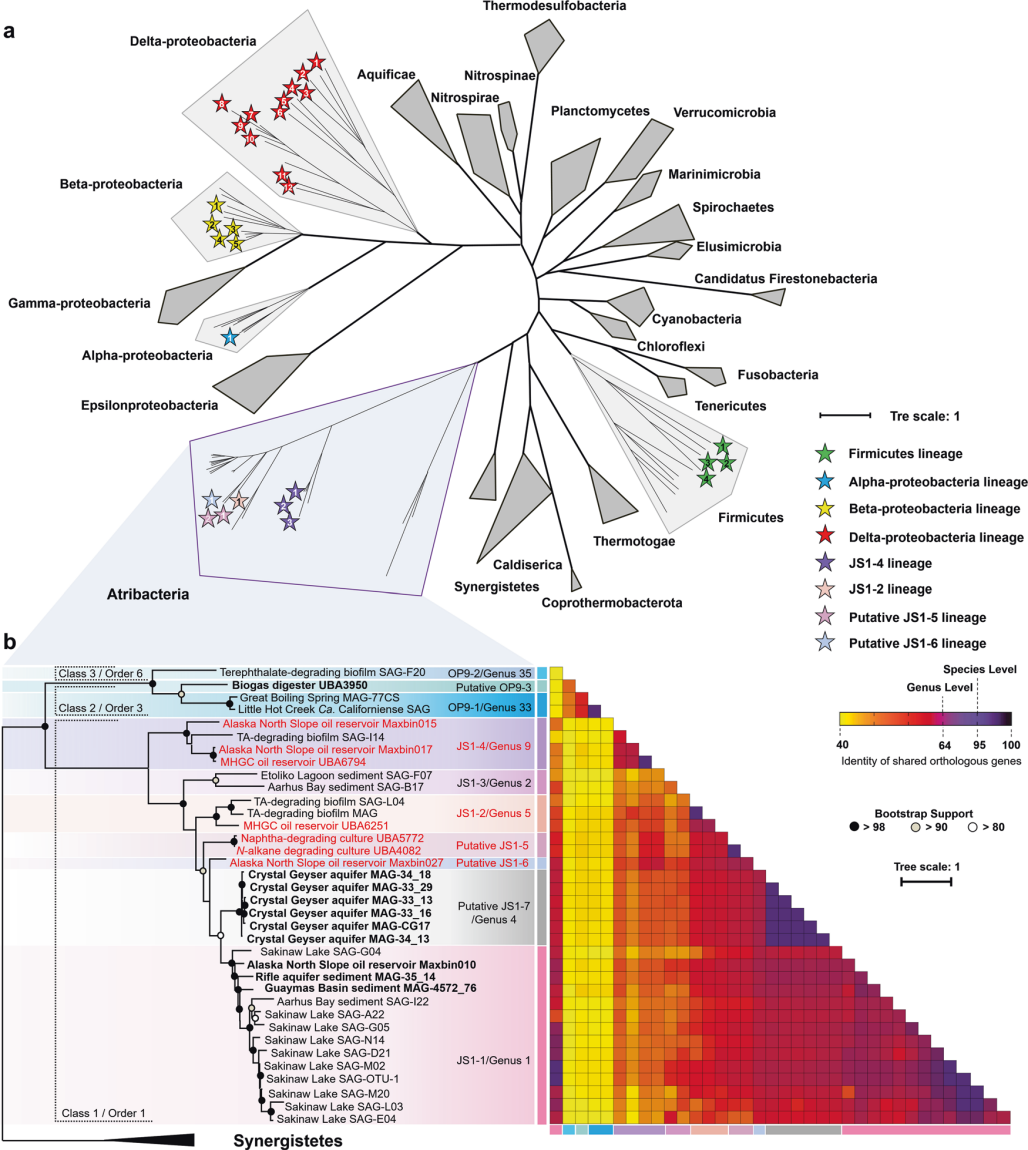
Phylogenomic analysis of atribacterial MAGs and SAGs with contamination less than 8%. **a** Phylogenomic placement of atribacterial genomes in a genome tree constructed with >200 reference archaeal and bacterial genomes. The tree was inferred from the concatenation of 400 conserved marker genes (Supplementary Table S4) [39]. Genomes containing at least one of the fumarate addition genes (*ass*/*bss*/*nms/hbS*) were indicated by stars, and the numbers inside the stars correspond to the taxonomic affiliation in Fig. 2a. **b** Detail phylogenetic relationships and heatmap of pair-wise orthologous similarity values of Atribacteria OP9 and JS1 as proposed by Nobu et al. [2]. Newly assembled and co-assembled MAGs were indicated in bold, MAGs containing FAE gene were indicated in red. Genes are designated as shared if they are bidirectional BLASTP hits (*E*-value <10^−5^). Branches that meet the Genus, Order and Class candidate taxonomic ranks proposed by Yarza et al. [1] are encompassed by dotted parentheses

Most of the Atribacteria-like MAGs in this study do not contain 16S rRNA gene sequences (Supplementary Fig. S1), and hence, the phylogeny of these MAGs was mainly inferred from genome trees and average AAI using standards for taxa descriptions of uncultivated microorganisms proposed recently (AAI: 45–65% for same family, 65–95% for same genus and 95–100% for same species) [51]. Eight out of seventeen newly assembled Atribacteria-like MAGs were classified into subgroups of JS1 and OP9 lineages proposed by Nobu [2] (Fig. 1a, b). Maxbin015 and Maxbin017 which were retrieved from metagenomes form an Alaska North Slope oil reservoir, together with UBA6791 which was obtained from MHGC oil reservoir metagenomes, formed a single clade with previously sequenced TA-degrading reactor biofilm SAG-I14 [8] and the pair-wise average AAI within them are all above 65% (Fig. 1b), indicating that they belonged to the same genus-level group as JS1-4. A 16S rRNA gene tree also placed Maxbin015 into the JS1-4 lineage, which corresponds to Genus_9 proposed by Yarza et al. [1] (Supplementary Fig. S1). Similarly, another MHGC oil reservoir MAG, UBA6251 showed a close relationship and high similarity (AAI of 75%) to TA-degrading biofilm SAG-L04, which suggested its affiliation to the JS1-2/Genus_5 lineage. MAGs from the Alaska North Slope oil reservoir (Maxbin010), hydrothermal sediments (MAG-4572_76), and Rifle sediment (MAG-35_14) were positioned in the JS1-1 group (or Genus_1) with a distinct relationship to previously reported SAGs from Sakinaw Lake and Aarhus Bay, indicating that these likely represent different species (Fig. 1b).

A near-full-length 16S rRNA gene sequence (1610 bp) was found in the Alaska North Slope oil reservoir Maxbin027, this sequence was classified into a divergent cluster distant from currently known taxonomic groups proposed by Yarza et al. [1, 2] and hence we propose a new taxonomic group of JS1-6 for Maxbin027.

Similarly, MAGs from short-chain *n*-alkane-degrading and naphtha-degrading enrichment cultures (UBA4082 and UBA5772, respectively) exhibited a close relationship in the genome tree and high AAI of 99%, suggesting that these two genomes were within a novel genus-level group of JS1-5 (Fig. 1b).

MAGs from Crystal Geyser aquifer metagenome, namely MAG-34_13, MAG-34_18, MAG-33_29, MAG-CG17, MAG-33_16 and MAG-33_13, represented a single species-level cluster with >95% AAI and 99–100% 16S rRNA gene identity (Fig. 1b and Supplementary Fig. S1). Phylogenetic analysis of the 16S rRNA gene sequences found in these MAGs demonstrated their affiliation to Genus_4. Here, we propose a new group of JS1-7 for these MAGs (Fig. 1b).

Only one MAG, UBA3950 that has been assembled from an anaerobic digester metagenome, was classified into the OP9 lineage. UBA3950 formed a divergent clade distinct from OP9-1 and OP9-2 clades (Fig. 1b), combined with the result that the average AAI between UBA3950 and other OP9 genomes were below the threshold for a genus level relationship (average AAI of 62%, Fig. 1b), UBA3950 was proposed to represent a novel genus-level group, OP9-3.

### Genes encoding fumarate addition enzymes in JS1 MAGs

**Fig. 2:**
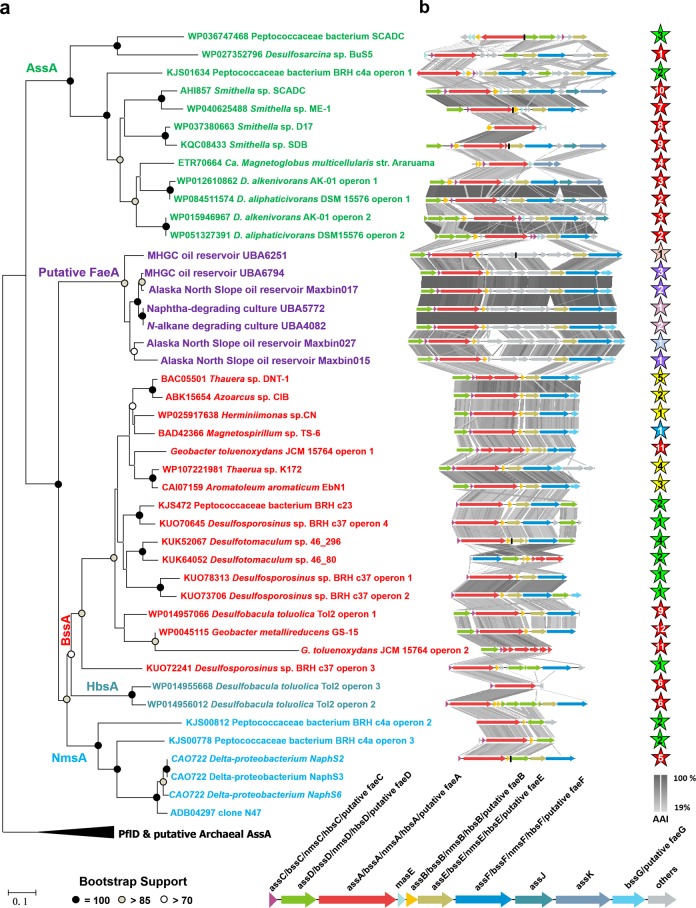
Analysis of FAE operons in atribacterial MAGs. **a** Phylogenetic analysis of Atribacterial *faeA* (861 amino-acid positions). **b** TBLASTx comparison of the FAE gene clusters of atribacterial MAGs with all other genomes containing fumarate addition enzymes. For the comparison, an *E*-value cutoff of 1e−10 was used, and visualization of the gene clusters was done with the program Easyfig [83]. In some genomes, the fumarate addition genes are located in separate contigs due to fragmentary assembly, and in these cases, borders between the single clusters are marked with black vertical lines. The stars on the right side correspond to the branch position of MAGs in Fig. 1a. A representative genomic organization of fumarate addition enzyme genes was shown at the bottom of figure

Sequences encoding putative alpha-subunit of FAE (*faeA*) were detected in five high-quality, Maxbin015, Maxbin017, Maxbin027, UBA5772 and UBA4082, and two medium-quality newly assembled MAGs, UBA625,1 and UBA6794 (Table 1), which represent lineages of JS1-4, JS1-2 and JS1-5 and JS1-6 as mentioned above. These *faeA* genes had very low identities to the top BLASTP hit of the *bssA* gene in *Desulfobacula toluolica* (YP_006759359, AAI around 55%) [24]. Phylogenetic study of these atribacterial *faeA* genes demonstrated a monophylogenetic and deeply branching cluster among fumarate addition genes (*assA*, *bssA*, *nmsA*, and *hbsA*), phylogenetically separate from the pyruvate formate lyase genes (*pflD*) (Fig. 2a).

Our analysis also revealed that the previously reported 2 copies of *assA* gene in Maxbin010 was most similar to *pflD* gene in *Moorella mulderi* (WP062284143, AAI: 38%) which is not known for anaerobic hydrocarbon degradation. The phylogenetic analysis of this gene also demonstrated its affiliation to *pflD* gene cluster (not shown in Fig. 2), and as a result, these genes were not considered as *faeA* homologs in this study. This result is expected since Maxbin010 was classified into JS1-1 lineage where no FAE-containing MAGs have been found, so far.

Apart from the putative alpha-subunit of FAE, genes encoding putative beta and gamma-subunits of FAE (*faeB* and *faeC*), a gene encoding an activating enzyme (*faeD*) and several genes of unknown function (*faeE*-*G*) were also detected in these MAGs [52]. These FAE-related genes clustered and formed an operon in contigs in atribacterial MAGs [53]. The two small subunits, namely FaeB and FaeC are unique for fumarate addition enzymes, and complete a heterotrimeric (αβγ)_2_ glycyl radical enzyme with a conserved Gly radical domain in the alpha-subunit, as well as binding sites for a [4Fe-4S] cluster in the beta- and gamma-subunits (Supplementary Fig. S2) [54]. The FaeD from these MAGs contains two cysteine clusters matching the typical ferredoxin consensus sequence motif “CxxCxxCxxxC” (Supplementary Fig. S3), which differs from the previously known activating enzymes for pyruvate formate lyase [55].

Phylogenetic analysis based on amino acid sequences of FaeB-G also demonstrated their association with fumarate addition genes rather than genes with other functions (Supplementary Fig. S4–9). A synteny analysis was performed on putative FAE genes in atribacterial MAGs in comparison with other fumarate addition genes, and the arrangement of *faeDCAB* in atribacterial operons was quite similar to canonical *bssDCAB* operons in most *bss*-containing genomes and *assDCAB* in *Smithella* genomes (Fig. 2b) [56]. Notably, insertion of genes of C4-dicarboxylate-binding protein (DctP) and C4-dicarboxylate transporter (DctQ and DctM) were observed in all atribacterial FAE operons. These proteins form an ATP-independent periplasmic (TRAP) membrane transporter specific for C4-dicarboxylate, likely succinate and fumarate [57].

### Genes involved in anaerobic pathways downstream of fumarate addition

**Fig. 3:**
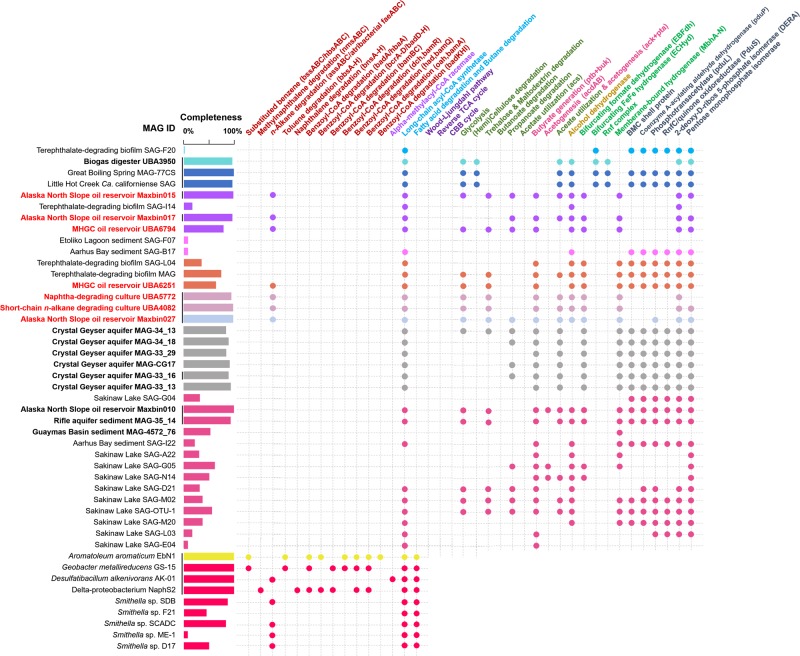
Metabolic pathways identified in atribacterial MAGs and SAGs with contamination less than 8%. Newly assembled and co-assembled MAGs were indicated in bold, and MAGs that contain fumarate addition enzymes were highlighted in red. Genome with completeness >90% that meet the rank of high-quality were underlined [26]. The colour code of the columns and dots in the igure are the same to the colour code of stars in Figs. 1a,  2b and the enzyme names were colored and grouped based on their functions. Pathways that have been previously described in Atribacteria lineages were considered as present when 75% of the genes involved in the pathway were detected. Pathways that have not been found in Atribacteria lineages before were defined as present only if all genes involved in the pathway were detected. Identification of the pathways was carried out with pathway mapping on both RAST server and KEGG server. Results were manually checked by BLASTP of the genes against NCBI-nr database. Isolated microorganisms were known to be capable of anaerobic fumarate addition to hydrocarbons were used as positive controls for identification of genes involved in anaerobic hydrocarbon degradation. Abbreviations are: *bssABC*, benzylsuccinate synthase; *nmsABC*, naphthyl-2-methyl-succinate synthase; *assABC* alkylsuccinate synthase; *bbsEF*, benzylsuccinate-CoA transferase; *bbG*, benzylsuccinyl-CoA dehydrogenase; *bbsH*, enoyl-CoA hydratase; *bbsCD*, 3-hydroxyacyl-CoA dehydrogenase; *bbsAB*; 3-oxoacyl-CoA thiolase; bnsEF, naphthyl-2-methyl-succinate-CoA transferase; *bnsG*, naphthyl-2-methyl-succinyl-CoA dehydrogenase; *bnsH*, naphthyl-2-methylene-succinyl-CoA hydratase; *bnsCD*, naphthyl-2-hydroxymethyl- succinyl-CoA dehydrogenase; *bnsAB*, naphthyl-2-oxomethyl-succinyl-CoA thiolase; *badA*, benzoate-CoA ligase; *hbaA*, 4-hydroxybenzoate-CoA ligase; *bcrA*-*D*; *acs*, acetyl-CoA synthetase; *pka*, phosphate acetyltransferase; *ack*, acetate kinase. A detail information of the genes in this figure could be found in Supplemenatary Table S5

Following the initial fumarate addition of alkyl-substituted benzene which leads to the formation of (R)-benzylsuccinate, benzylsuccinate is converted to benzoyl-CoA through *β*-oxidation-like reactions [58], and genes encoding for the enzymes involved are *bbsA*-*G*. However, no homologs of *bbsA*-*G* were found in atribacterial genomes (Fig. 3 and Supplementary Table S5). *BnsA*-*G* genes, which encode for the enzymes involved in *β*-oxidation reactions converting naphthyl-2-methyl-succinate to 2-naphthoyl-CoA, were not detected in these MAGs, either. It has been proposed that reductive de-aromatization of benzoyl-CoA can occur, either via an ATP-dependent benzoyl-CoA reductase found in facultative organisms (*bcrA*-*D*), or via an ATP-independent reductase found in strict anaerobes (*bamB*-*I*) [59]. Further degradation of this compound resembles a modified *β*-oxidation pathway, the genes associated with the sequential reactions are *Dch*/*BamR*, *had*/*bamQ* and *oah*/*BamA* for *Thauera*-type *β*-oxidation, or *BadKHI* for *Rhodopseudomonas*-type *β*-oxidation [59]. Searches for these genes were performed, only annotations of *bcrBCD* were found in some of the atribacterial MAGs using the RAST server. However, further analysis of these *bcrBCD* genes demonstrated top BLASTP hits to 2-hydroxyglutaryl-CoA dehydratase (*hgdABC*) from microorganisms not known for anaerobic aromatic compound degradation [60].

By contrast, genes required for alkane degradation following fumarate addition, inclusive of CoA-synthetase/ligase (*assK*) for CoA activation, a putative methylmalonyl-CoA mutase (*mcmLS*) for carbon skeleton rearrangement and a putative propionyl-CoA carboxylase (*pccA*) (formerly referred to as methylmalonyl-CoA carboxyl transferase, *mcd*) [61], were found in all MAGs containing the *fae* genes except for a putative alpha-methylacyl-CoA racemase for epimerization of methylalkylsuccinic acids [61] (Supplementary Table S5).

The pathways for benzoyl-CoA and alkanes degradation converge at a “conventional” *β*-oxidation pathway. However, the genes for *β*-oxidation were largely absent in atribacterial MAGs, which is consistent with previous studies [2, 10].

### Other metabolic potential of newly assembled MAGs

In this study, the presence of genes found in all medium-to-high-quality MAGs/SAGs was taken as support for the presence of such gene in the represented lineages. To avoid false negatives caused by low genome completeness, however, only high-quality genomes were used when considering whether a gene is absent in the represented lineages proposed by Nobu et al. [2] and in this study.

**Fig. 4:**
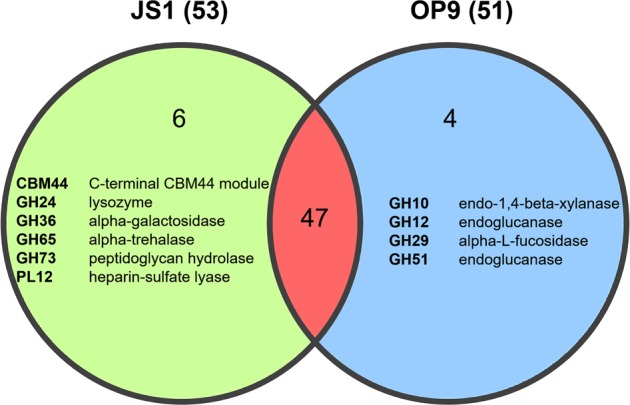
Carbohydrate-Active enZymes (CAZymes) identified in JS1 and OP9 pan-genomes. Genes that are specific to JS1 and OP9 were listed and coloured in green and blue, respectively. Genes that are common to both lineages were coloured in red. The total CAZy gene numbers in JS1 and OP9 were indicated in parentheses. A detail information of the genes in this figure could be found in Supplementary Table S6

By this criterion, capacity for autotrophy was analyzed for these newly assembled MAGs, genes for carbon fixation pathways were searched, but none of the MAG contained any pathways (Fig. 3). Most of the newly assembled MAGs and several SAGs published previously [2], which represent OP9-3, JS1-1, JS1-2, JS1-4, JS1-5, JS1-6, and JS1-7 lineages contain a suit of genes encoding glucokinase, glucose-6-phosphate isomerase, 6-phosphofructokinase, diphosphate-dependent phosphofructokinase, fructose-bisphosphate aldolase, glyceraldehyde 3-phosphate dehydrogenase, phosphoglycerate kinase, 2, 3-bisphosphoglycerate-cindependent phosphoglycerate mutase, enolase, pyruvate kinase, which comprise the whole Embden–Meyerhof glycolysis pathway (Fig. 3 and Supplementary Table S5). Pyruvate ferredoxin oxidoreductase and 2-oxoacid oxidoreductase (ferredoxin), which convert pyruvate generated from glycolysis into acetyl-CoA were also found in these MAGs. UBA3950, representing the OP9-3 lineage encodes putative glycohydrolases and an endoglucanase that may enable catabolism of (hemi)cellulose (Fig. 3), which is consistent with a previous study of “*Ca*. Caldatribacterium californiense” and “*Ca*. Caldatribacterium saccharofermentans” in OP9-1 [10]. MAGs classified here within designated JS1 lineages lack such genes but have putative alpha-trehalase and alpha-galactosidase that could enable utilization of trehalose and galactose, as revealed by pan-genomics of JS1 and OP9 (Fig. 4 and Supplementary Table S6). Genes encoding phosphate butyryltransferase (*ptb*) and butyrate kinase (*buk*), which catalyze the reversible reaction of degrading butanoate into butanoyl-CoA and butanoate formation, were found in most JS1 MAGs. Further analysis demonstrated that these MAGs lack a complete butanoate degradation pathway, therefore, a putative role of butanoate generation was proposed for *ptb*+*buk* in these MAGs. The previous studied propionate degradation pathway in JS1-1 and JS1-2 lineages [2] was also found in several of the newly assembled JS1-1, JS1-4, JS1-6, and JS1-7 MAGs, but not in JS1-5 or OP9-3 MAGs. Genes of *ack* and *pta* were detected in OP9-3, and JS1 MAGs. Enzymes encoded by *pta*-*ack* genes could also catalyze acetogenesis from acetyl-CoA in the reverse reaction of acetate oxidation, and produce ATP at the same time [62]. Unexpectedly, Maxbin010, SAG-G05 and SAG-N14 that represent JS1-1 lineage possessed another acetyl-CoA synthetase (ADP-forming) (*acdAB*), this enzyme is mainly distributed in archaeal genomes [62]. Aldehyde dehydrogenases and alcohol dehydrogenases were found in OP9-3, and multiple JS1 MAGs except for JS1-7 MAGs. An electron-confurcating FeFe hydrogenase (ECHyd) was found in OP9-3 MAG, and an electron-bifurcating formate dehydrogenase (EBFdh) was found in multiple JS1 MAGs.

In a previous study, a chemiosmotic membrane potential was predicted to be maintained mainly by an rnf complex inferred from OP9 single cell genomics [10]. The Rnf complex is a membrane-bound, six-subunit complex capable of coupling NADH:Fd oxidoreductase activity with H^+^ or Na^+^ transport [63]. In this study, UBA3950 which represents a novel lineage of OP9-3 also contain this complex. In JS1-1 and JS1-2 lineages, a membrane-bound hydrogenase (MbhA-N) was proposed to couple Fd_red_ generation and H_2_ generation [2]. This complex was also present in most newly assembled MAGs of JS1 lineages.

Previous results showed that atribacterial members of OP9 and JS1 lineages found in Sakinaw Lake, Aarhus Bay, and a TA-degrading bioreactor contain putative bacterial microcompartment (BMC) shell proteins [2]. There are additional genes, such as PduP (coenzyme A-acylating aldehyde dehydrogenase), PduL (phosphotransacetylase), PduS (RnfC/quinone oxidoreductase (Nqo)-like NADH dehydrogenase), 2-deoxy-d-ribose 5-phosphate aldolase (DERA) and pentose monophosphate isomerase within the BMC cluster, suggesting a link between BMC and aldehyde and sugar metabolism in Atribacteria [2]. Although we also found the BMC clusters in multiple newly assembled MAGs representing JS1-1, JS1-2 and JS1-7 lineages, we failed to detect BMC shell proteins in high-quality MAGs of OP9-3 (UBA3950), JS1-4 (Maxbin015, Maxbin017, and UBA6794), JS1-5 (UBA5772 and UBA4082) or JS1-6 (Maxbin027) (Fig. 3).

## Discussion

### Phylogenetic diversity of newly assembled MAGs

We expanded genomic coverage of the Atribacteria phylum (OP9/JS1) by classifying ten MAGs into three novel genus-level JS1 lineages, namely JS1-5, JS1-6, and JS1-7, and one novel OP9 lineage, OP9-3 (Fig. 1b). We also classified seven newly assembled Atribacteria-like MAGs into currently known lineages of JS1-1, JS1-2, and JS1-4. Within these MAGs, Maxbin015, Maxbin017, Maxbin010, and MAG-35_14 are the only high-quality genomes that represent JS1-1 and JS1-4 lineages known so far. Previous genomic studies have uncovered metabolic potential of OP9-1, JS1-1, and JS1-2, however, detailed study of JS1-3 and JS1-4 was hampered due to the extremely low genome completeness of single cell genomes [2]. The newly assembled high-quality and medium-quality MAGs reported in this study enable metabolic insight into the previously unknown lineages of JS1-4, JS1-5, JS1-6, JS1-7 and OP-3, and also enlarge the current understanding about JS1-1 and JS1-2 lineages.

### Incomplete anaerobic hydrocarbon degradation

Complete FAE operons, found in the newly assembled high and medium quality MAGs, expands the current knowledge of the Atribacteria phylum [2, 10]. The FAE-containing MAGs are broadly distributed within JS1-2, JS1-4, JS1-5, and JS1-6 lineages, which were all retrieved from samples associated with hydrocarbon-enriched environments where biodegraded oil has been observed [21, 64, 65], and hydrocarbon degradation enrichment cultures [5, 20, 22, 29]. Thus, we posit a potential anaerobic hydrocarbon degrading role for members from these lineages. Furthermore, except for Maxbin010 that came from Alaska North Slope oil reservoir and MAG-4572_76 that came from Guaymas Basin where hydrocarbons are abundant [34], the non-FAE-containing JS1 MAGs/SAGs mainly inhabit groundwater (Crystal Geyser aquifer [32]), brackish waters (Sakinaw Lake [8]), marine sediments (Aarhus Bay [11]) and aquifer sediments (Rifle [33]) where no impact of hydrocarbons has been reported, indicating that capacity of anaerobic hydrocarbon degradation may not be a common character for JS1 members in non-hydrocarbon-impacted environments.

It is noteworthy that the atribacterial *fae* genes formed a monophylogenetic clade distantly related to other known *fae* genes in phylogenetic trees constructed from amino acid sequences, hinting at a substrate specificity which may be different from other fumarate addition-catalyzing enzymes studied so far. The inserted genes in atribacterial FAE operons that encode transport system for succinate and fumarate may potentially participate in initial hydrocarbon activation, probably utilizing an exogenous fumarate for hydrocarbon activation.

The downstream pathways following fumarate addition of aromatics were absent in these FAE operon-containing MAGs and other atribacterial genomes sequenced, so far, which do not support a degradation capacity for aromatics in these MAGs [66]. However, due to the novelty of JS1 lineages, the enzymes involved in these pathways would be difficult to predict. By contrast, genes associated with pathways downstream of fumarate addition were conserved in these MAGs except for alpha-methylacyl-CoA racemase, indicating a potential capacity for anaerobic *n*-alkane degradation in these JS1 members. We also noted that alpha-methylacyl-CoA racemase was also missing in all *n*-alkane-degrading *Smithella* draft MAGs which have been obtained from *n*-alkane-degrading enrichment cultures (Fig. 3) [53, 67], suggesting that this gene may not be necessary for anaerobic *n*-alkane degradation in these microorganisms. Although the epimerization of CoA-activated (1-methylpentyl)succinate was proposed in a study of addition of C2 of long-chain alkanes to fumarate [67–69], it has so far not been studied in addition of C1 of short-chain alkanes, such as propane and butane, to fumarate [70]. The potential ability to degrade short-chain *n*-alkane explains the wide distribution of FAE-containing MAGs in oil reservoir and oilsands tailing ponds where short-chain *n*-alkanes are readily available as major constituents of crude oil, as well as short-chain *n*-alkane-degrading (C6-C10) and Naphtha-degrading enrichment cultures where short-chain *n*-alkanes are the major substrate [29].

**Fig. 5:**
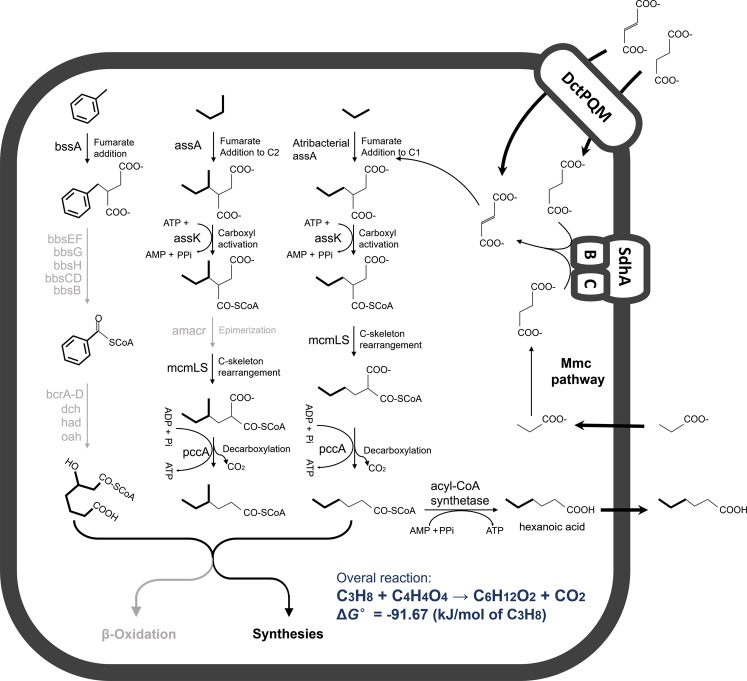
Anaerobic hydrocarbon degradation inferred from JS1 MAGs associated with hydrocarbon-enriched environments. Pathways with genes detected were depicted in solid black lines, pathway with genes not found in all MAGs were depicted in grey lines. The ATP production/consumption in putative reaction catalyzed by *assK* and *pccA* were predicted resembling well-defined reaction of acetyl-CoA ligation and methylmalonyl-CoA decarboxylation. Δ*G*° standard Gibbs free energy was calculated based on reactants and products at 1 M concentration and gases at a partial pressure of 1atm. Gibbs free energy of formation for fumarate in the liquid state was taken from NIST Chemistry WebBook (https://webbook.nist.gov/chemistry/), all other compounds are in the aqueous phase

No evidence for fatty acid degradation has been found in taxa from the Atribacteria phylum [2, 10] and therefore, we proposed a fermentative metabolism of short-chain *n*-alkane for these MAGs, in which short-chain *n*-alkanes like propane could be converted to acyl-CoA through C1 addition to fumarate, carbon-skeleton rearrangement and decarboxylation, followed by fatty acid formation (Fig. 5). The produced fatty acids could be metabolized by other heterotrophic bacteria in microbial consortia, such as the most abundant *Desulfotomaculum* organisms in the Alaska North Slope oil reservoir [22], or the most dominant Firmicutes-related OTUs (*Desulfotomaculum* and *Desulfosporosinus*) in the NAPDC culture and Deltaproteobacteria in the SCADC culture [30]. In return, propionate could be produced by these partner organisms as a fermentation product in environments where relatively low levels of inorganic electron acceptors for use in anaerobic respiration are available [2]. The produced propionate is a potential source of fumarate for Atribacteria organisms since fumarate is not recycled in the fumarate addition pathway in Atribacteria. Alternatively, fumarate/succinate could be supplied exogenously from outside directly through transporter which is encoded in the FAE operon. Fumarate and succinate are important intermediates in the citric acid cycle, and the co-occurring Firmicutes and Deltaproteobacteria [22, 29] which contain complete or partial citric acid cycle pathway could be potential suppliers of fumarate/succinate [71]. In this hypothesized pathway, Atribacteria organisms would gain one molecule of ATP by fermentation of one alkane molecule (Fig. 5). The incompletely degraded products could serve as precursors for fatty acid biosynthesis (Fig. 5), especially when considering the predicted heterotrophic lifestyle of Atribacteria [2, 10]. However, this hypothesized short-chain *n*-alkane metabolism pathway in this study requires further evidence, especially using stable isotope probing techniques combined with metabolite detection in enrichment cultures [72, 73].

### Expanded metabolic capacities of OP9 and JS1 lineages

We found no evidence for inorganic carbon fixation pathways in these MAGs, indicating a heterotrophic lifestyle for Atribacteria [10]. In contrast to previous result that Atribacteria members from the JS1-1 and JS1-2 lineages were predicted to be incapable of carbohydrate fermentation [2], all genes required for the Embden–Meyerhof glycolysis pathway were found in multiple MAGs from JS1 lineages. Hitherto, the ability of carbohydrate degradation now extends from OP9 lineages (OP9-1, OP9-2, and OP9-3) to JS1 lineages (JS1-1, JS1-2, JS1-4, JS1-5, JS1-6, and JS1-7). Interestingly, genomic evidence showed a different sugar specialization for JS1 and OP9, based on which OP9 members are predicted to metabolize (hemi)cellulose and JS1 members are potential degraders of trehalose and galactose [74]. Ability to metabolize trehalose is broadly distributed within the bacterial phyla, as it is an important source of energy [75].

Genes for propionate degradation were still restricted in JS1 lineages. Members of JS1 lineages have the potential to gain ATP through butanoate generation, and acetate could serve either as carbon source or as end product during fermentation in lineages of OP9 and JS1. Acetyl-CoA synthetase (ADP-forming) (ACD, encoded by *acdAB*) have been regarded as being specific for Archaea, until recently, several *acdAB* homologies were found in bacterial genomes [76, 77]. The detection of *acdAB* genes in JS1-1 members unraveled an alternative ATP generating pathway coupled to acetate production in JS1-1 in addition to *ack*+*pta*, which has not been reported so far. Consistently, Atribacteria are frequently abundant in anoxic methane-rich sediments and have been suggested to have a potential role in methanogenesis by providing methanogenic Archaea with substrates such as acetate and CO_2_ [5, 17, 78].

Reduced electron carriers, such as NADH and reduced ferredoxin (Fd_red_) generated from substrate oxidation need re-oxidation. OP9 and JS1 members may consume reducing power by producing alcohol (aldehyde and alcohol dehydrogenases) and/or H_2_ (NiFe hydrogenase and FeFe hydrogenase). In addition, both NADH and Fd_red_ could be concomitantly oxidized through an electron-confurcating hydrogenase in OP9 or through an electron-bifurcating formate dehydrogenase in JS1 as a strategy for facilitating thermodynamically limited syntrophic catabolism [79, 80]. In OP9 lineages, the excess Fd_red_ is proposed to drive Rnf-mediated energy conservation in the membrane [2] in JS1 lineages, re-oxidation of excess Fd_red_ could be coupled to H_2_ generation by membrane-bound hydrogenase (Mbh). Therefore, a close syntrophic relationship between Atribacteria and other formate-consuming or H_2_-consuming microorganisms that depend on formate or H_2_ transfer is required to relieve thermodynamic constraints [81].

Nobu *et al*. proposed that the BMC cluster is an ancestral trait within the Atribacteria phylum (OP9 and JS1), and BMC-mediated aldehyde conversion to sugars is central to Atribacteria metabolism [2]. However, this gene cluster was not found in OP9-3, JS1-4, JS1-5, and JS1-6 MAGs with high completeness, indicating that BMC-associated metabolism may not be important in members of these lineages.

## Conclusion

The current understanding of the diversity, biology and ecology of candidate phylum “Atribacteria” (OP9/JS1) is very limited, especially considering that many of these enigmatic lineages are predominant in some environments. In this study, we classified several medium-to-high-quality Atribacteria-like MAGs into currently known lineages of JS1-1, JS1-2, JS1-4 and four novel genus-level groups of JS1-5, JS1-6, JS1-7, and OP9-3. In the light of comparative genomic analysis, members of JS1-2, JS1-4, JS1-5 and JS1-6 lineages from hydrocarbon-enriched environments were predicted to degrade short-chain *n*-alkanes, which could be broken down into fatty acids and then consumed by secondary degraders in the community or re-directed into fatty acid biosynthesis. This study proposes Atribacteria (JS1-2, JS1-4, JS1-5 and JS1-6) to be the third phylum of which members contain the complete suite of fumarate addition genes in addition to Proteobacteria (alpha/beta/gamma) and Firmicutes. Furthermore, OP9-3 members are also predicted to be (hemi)cellulose degraders and JS1 members are predicted to specialize in trehalose and galactose degradation. In JS1 lineages, the metabolism of sugar and organic acids, such as propionate could be coupled with butanoate/acetate/ethanol production, or through syntrophic metabolism by producing formate/H_2_. Collectively, these potential metabolisms highlight the important ecological role for the “Atribacteria” in the global carbon cycle, especially in hydrocarbon-enriched environments, such as oil reservoirs [82], methane hydrates [16], and hydrocarbon seeps where this candidate phylum is frequently detected.

## Supplementary information

Supplementary File S4

Supplementary Information

Supplementary Figure S3

Supplementary Table S2-4

Supplementary Table S5

Supplementary Table S6

Supplementary File S1

Supplementary File S2

Supplementary File S3
