# Supplementary material for: Anaerobic hydrocarbon degradation in candidate phylum ‘Atribacteria’ (JS1) inferred from genomics
Source: ISME J. 2019 Jun 6;13(9):2377–90. doi: 10.1038/s41396-019-0448-2 (PMC6776118; doi:10.1038/s41396-019-0448-2)
Supplement: Supplementary file 2 — Supplementary Information [file 41396_2019_448_MOESM2_ESM.docx]

**Supplementary materials**

***The ISME Journal***

**Anaerobic hydrocarbon degradation in candidate phylum ‘Atribacteria’ (JS1) inferred from genomics**

**Supplementary Information**

**Supplementary Methods**

*Phylogenetic analysis of 16S rRNA genes*

To construct 16S rRNA gene phylogenetic tree, 16S rRNA genes were first predicted by RNAmmer using the scaffolds from MAGs as queries [1], then complemented and verified by searching annotation of 16S rRNA gene in MAGs on the RAST server [2]. Sequences longer than 500 bp were used as queries and searched for similar sequences using BLASTN on NCBI website. The reference sequences with top hit to queries were downloaded and aligned together with query sequences using MAFFT with iterative refinement methods ‘Q-INS-i’ [3]. Alignments were then refined on Gblock server with strict selection settings [4]. Maximum likelihood tree was constructed and viewed in MEGA7 with 1000 bootstrap replicates [5].

*Comparison of FaeABC sequence active site conservation and conformation*

The predicted tertiary structure of FaeA, FaeB, and FaeC from the MAG-Maxbin017 genomes was predicted using I-TASSER v5.0 with default parameters [6]. Because there is only BSS model available in the database so far, the best fits for these structures were BSS models representing fumarate-adding enzymes. C-score of the top models for BssA, BssB, and BssC were 1.38, -0.09 and -0.74 respectively. Protein-ligand binding sites were verified with the COACH package embed in the I-TASSER web service[6]. Thauera aromatica crystal structure (PDB ID: 4PKF) was the best fit to the Bss complex in Bin 16 according to the result from I-TASSER. Individual predicted model of BssA, BssB, and BssC were superimposed on the 4PKF model using TM-align[7]. For BssA, BssB, and BssC, the TM-score for the alignment to the 4PKF structure were 0.961, 0.794 and 0.706 respectively.

**Supplementary Figures**


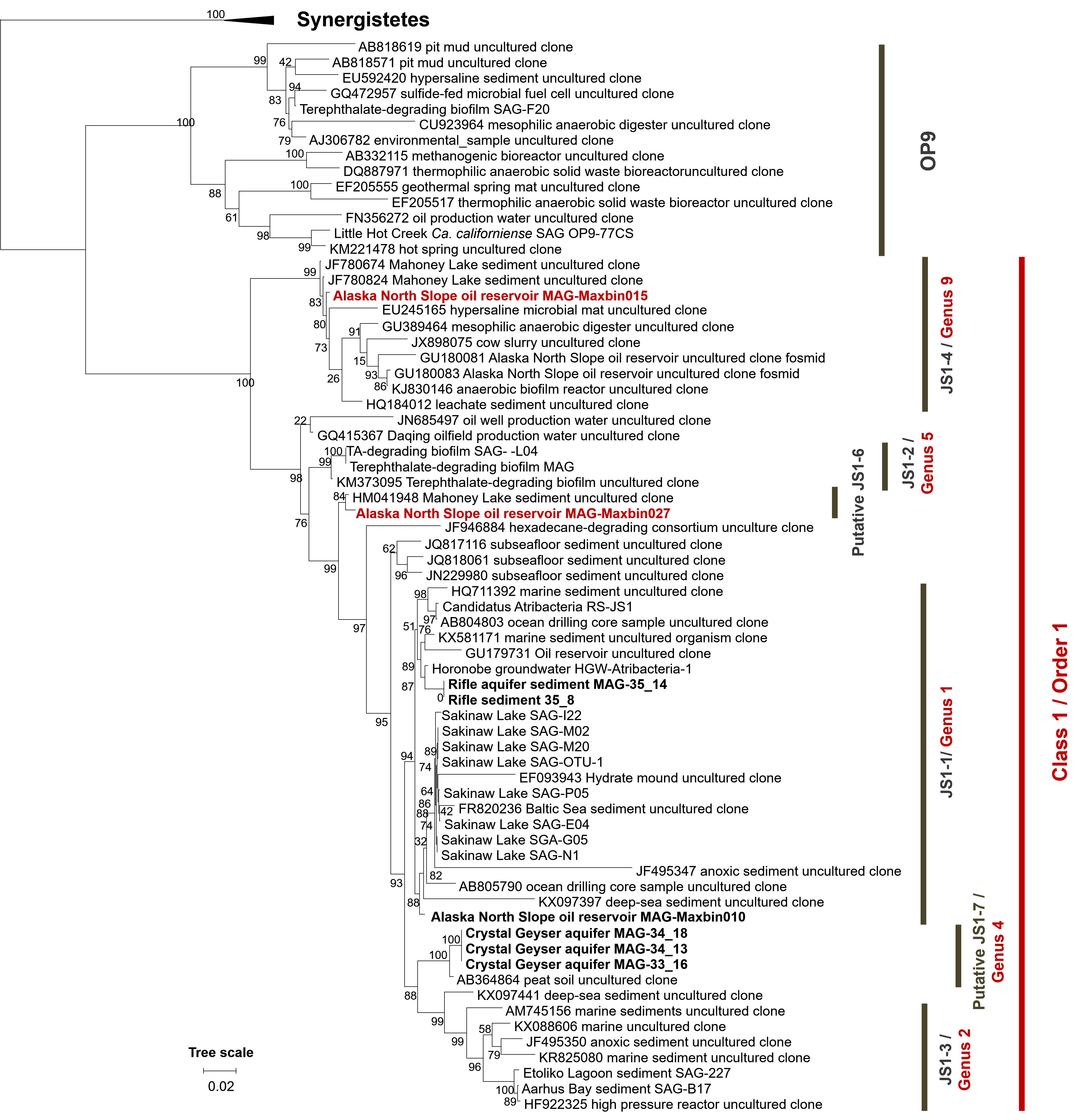


**Figure S1. Phylogenetic analysis of 16S rRNA sequences from MAG-Maxbin015 and reference sequences.** Maximum likelihood tree was constructed with 1000 bootstrap replicates. 16S rRNA gene sequences of MAG-Maxbin015 and MAG-Maxbin027 were highlighted in red. The region of 16S rRNA gene sequences used for this alignment corresponded to positions 101-1481 in the 16s rDNA nucleotide sequence of *E*. coil (GeneBank: AE014075.1). Genus, Order and Class candidate taxonomic units proposed by Yarza *et al.* [8] that encompass the JS1 data sets are indicated in red, and JS1 lineages proposed by Nobu *et al.* [9] and putative lineages proposed in this study are indicated in black.


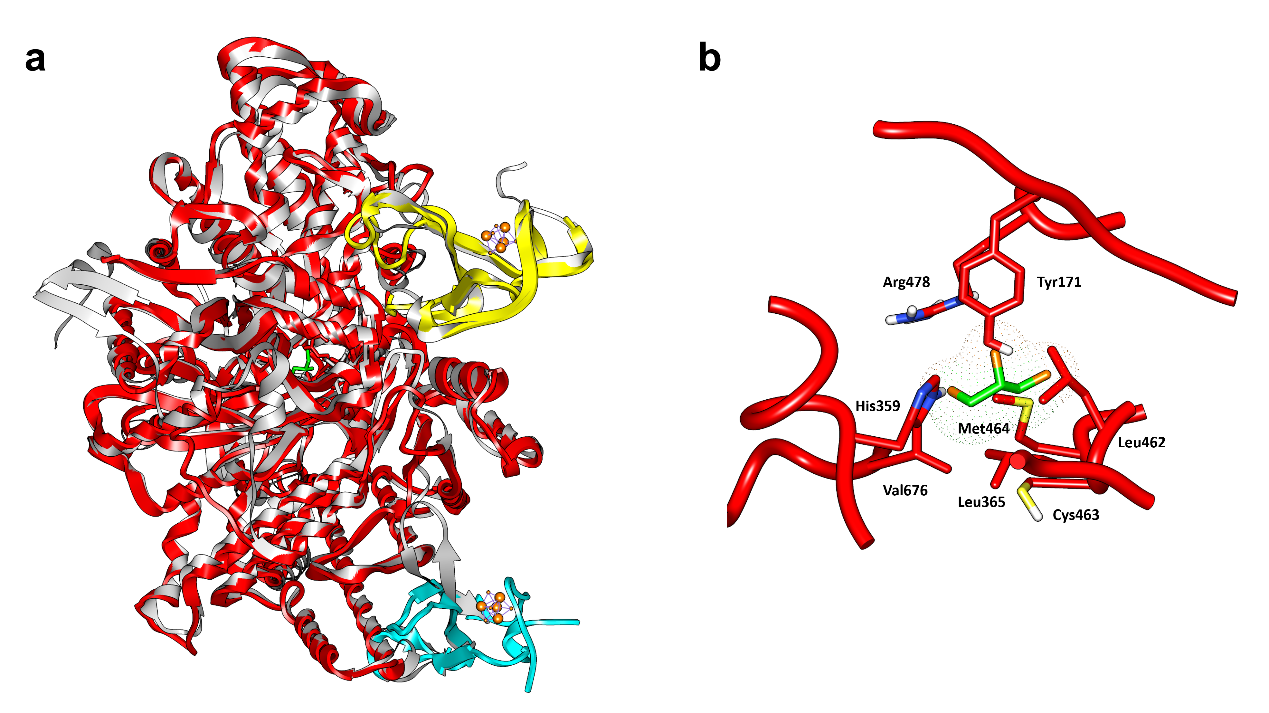


**Figure S2.** **Structural modelling and active sites of the FaeABC complex in MAG-Maxbin017**

Models of the FaeA (red), FaeB (yellow) and FaeC (cyan) in MAG-Maxbin017 aligned with BssABC (silver) of Thauera aromatica strain T1 crystal structure (PDB ID: 4PKF). The TM-score for the alignments in protein structure were high between the MAG-Maxbin017 and Thauera aromatica crystal structures (FaeA: 0.961, FaeB: 0.794, FaeC: 0.706). b, Model of the active sites of the FaeA (red) of MAG-Maxbin017 superimposed onto BssA (silver) of Thauera aromatica strain T1. Backbones of the complex in close proximity to ligand binding sites are shown. [4Fe-4S] cluster (orange), Gly radical domain (green) for MAG-Maxbin017 that correspond to cofactor ligand binding sites are shown with interacting amino acid side chains

**Figure S3. Sequence alignment of activating enzyme for FAE identified in this study with reference sequences.** Conserved residues are highlighted, motif ‘CxxCxxCxxxC’ are marked as red arrows.


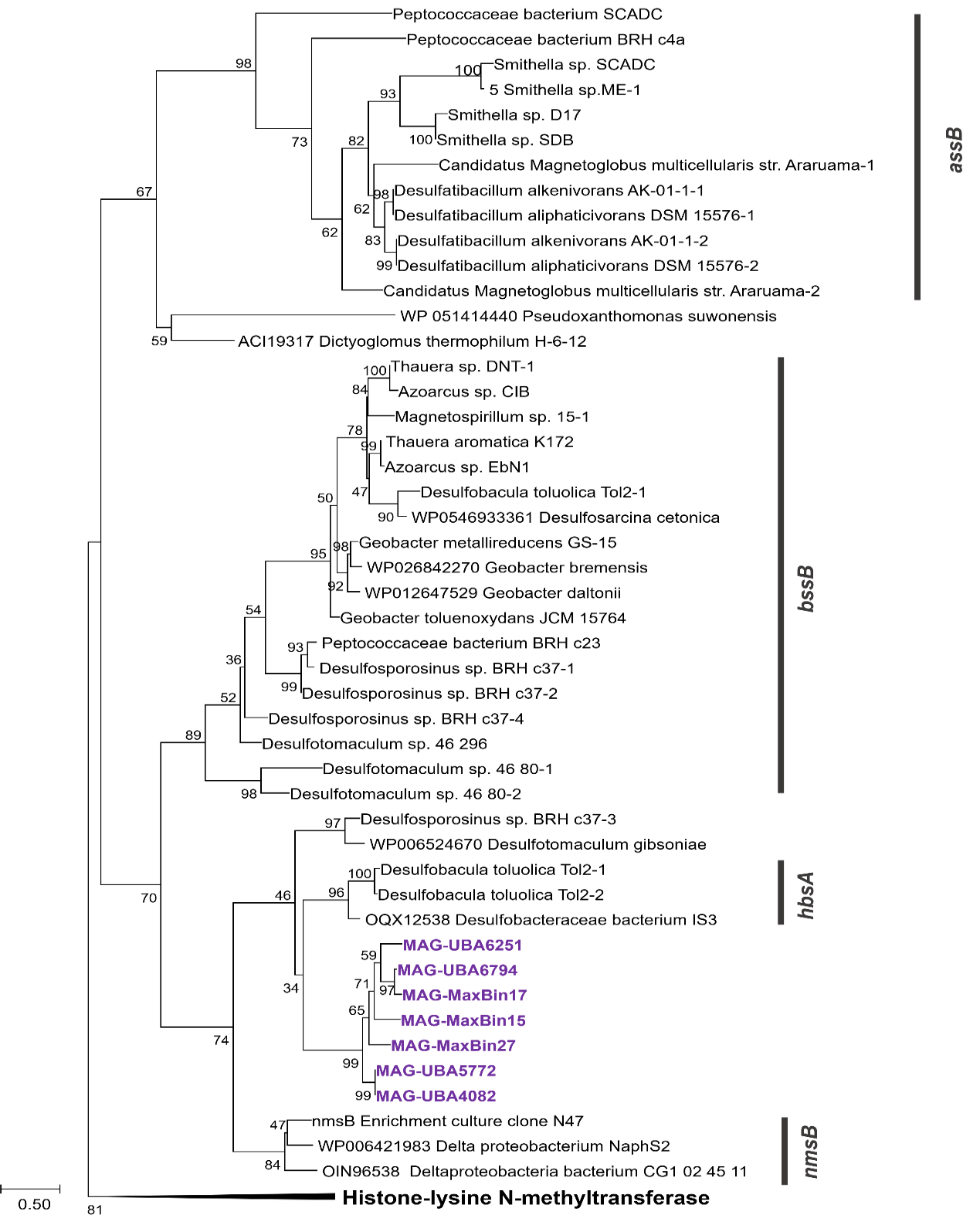


**Figure S4. Phylogenetic analysis of beta-subunit of fumarate-adding enzymes.** Sequences retrieved from Atribacterial MAGs were highlighted in purple. Maximum-likelihood tree were constructed in IQTREE Web Server with ‘standard’ model. Bootstrap values were determined using non-parametric bootstrapping with 1000 replicates and are shown on the tree.


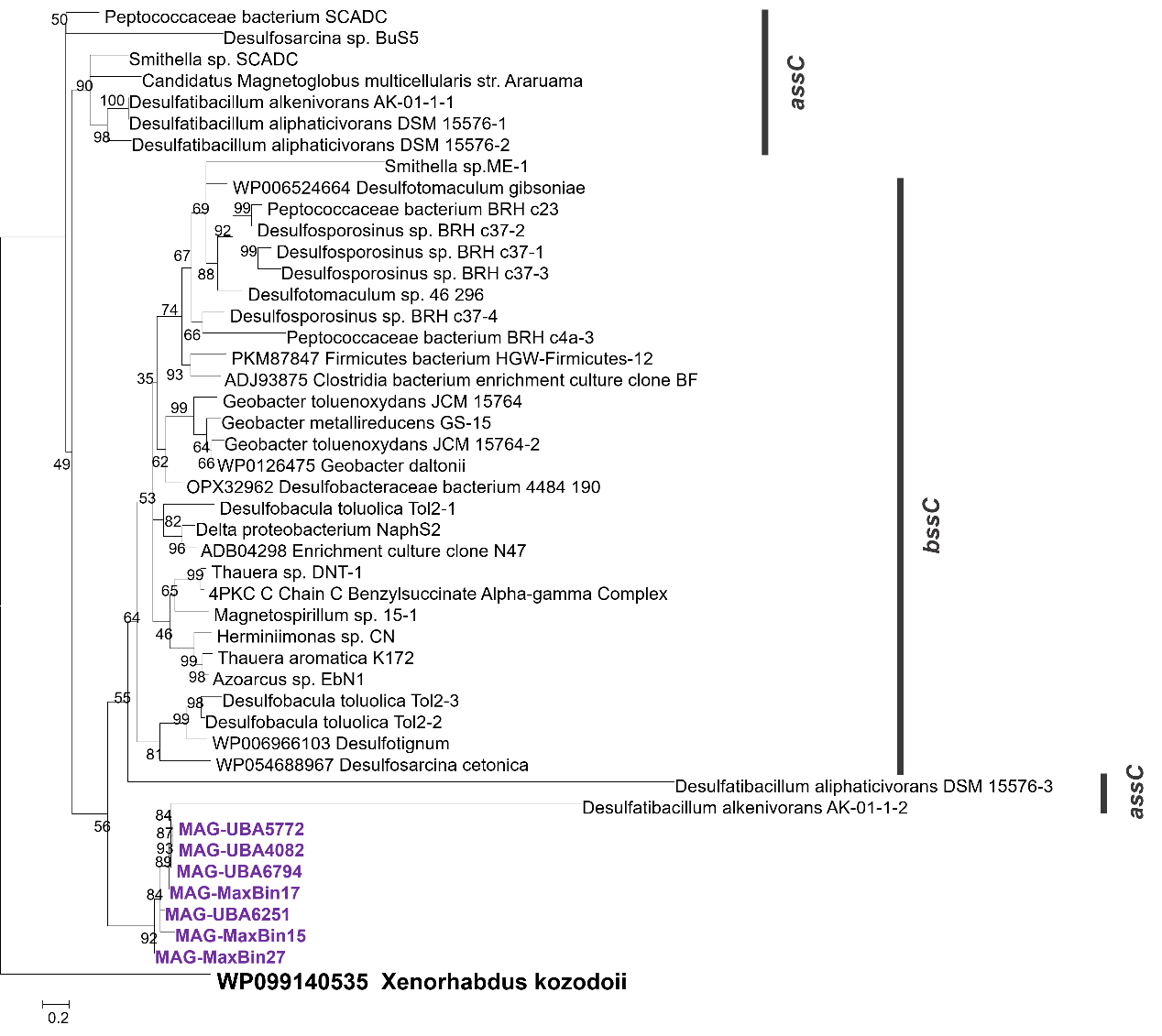


**Figure S5. Phylogenetic analysis of gamma-subunit of fumarate-adding enzymes.** Sequences retrieved from Atribacterial MAGs were highlighted in purple. Maximum-likelihood tree were constructed in IQTREE Web Server with ‘standard’ model. Bootstrap values were determined using non-parametric bootstrapping with 1000 replicates and are shown on the tree.


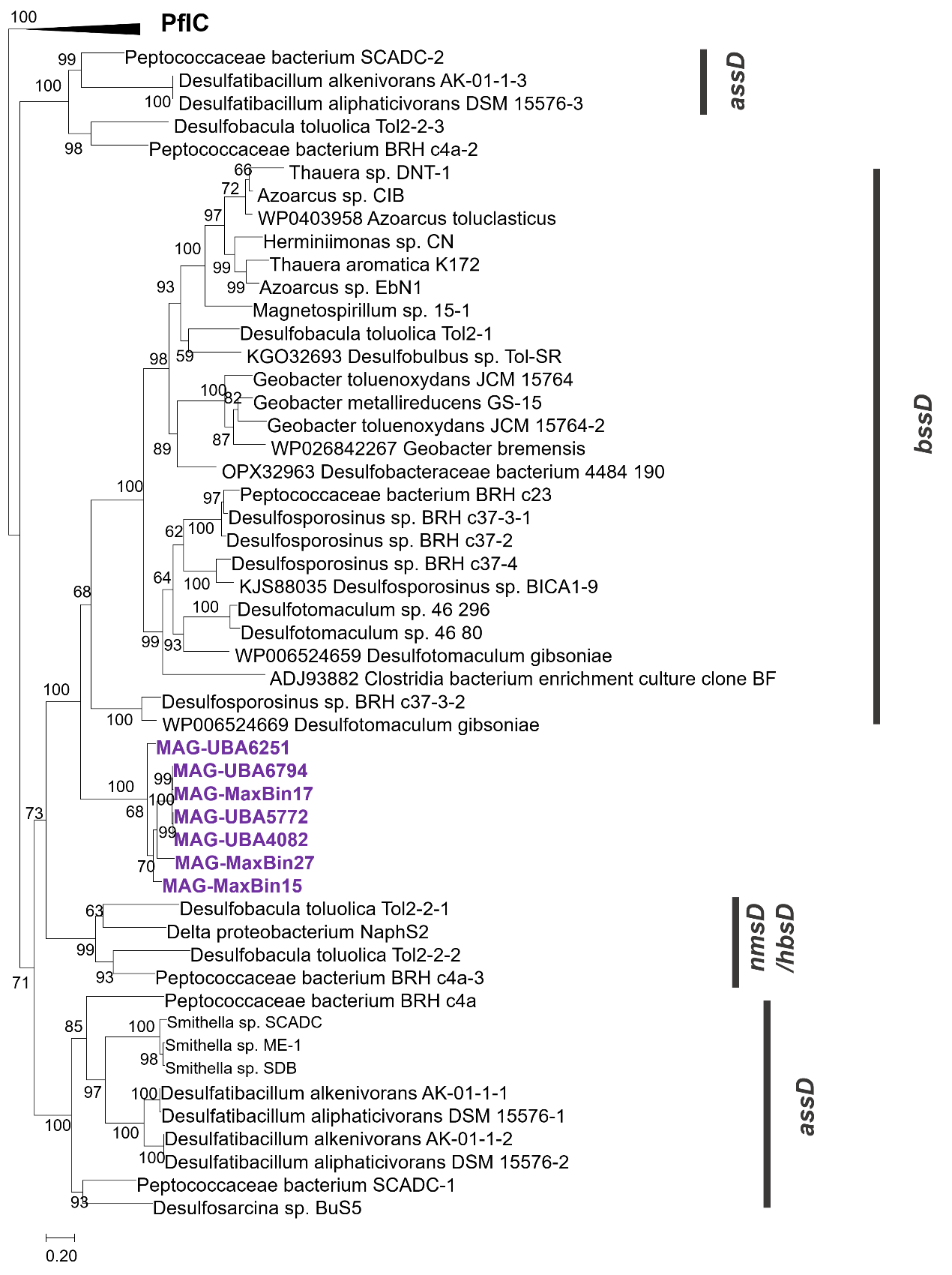


**Figure S6. Phylogenetic analysis of activating enzymes for fumarate-adding enzymes.** Sequences retrieved from Atribacterial MAGs were highlighted in purple. Maximum-likelihood tree were constructed in IQTREE Web Server with ‘standard’ model. Bootstrap values were determined using non-parametric bootstrapping with 1000 replicates and are shown on the tree.


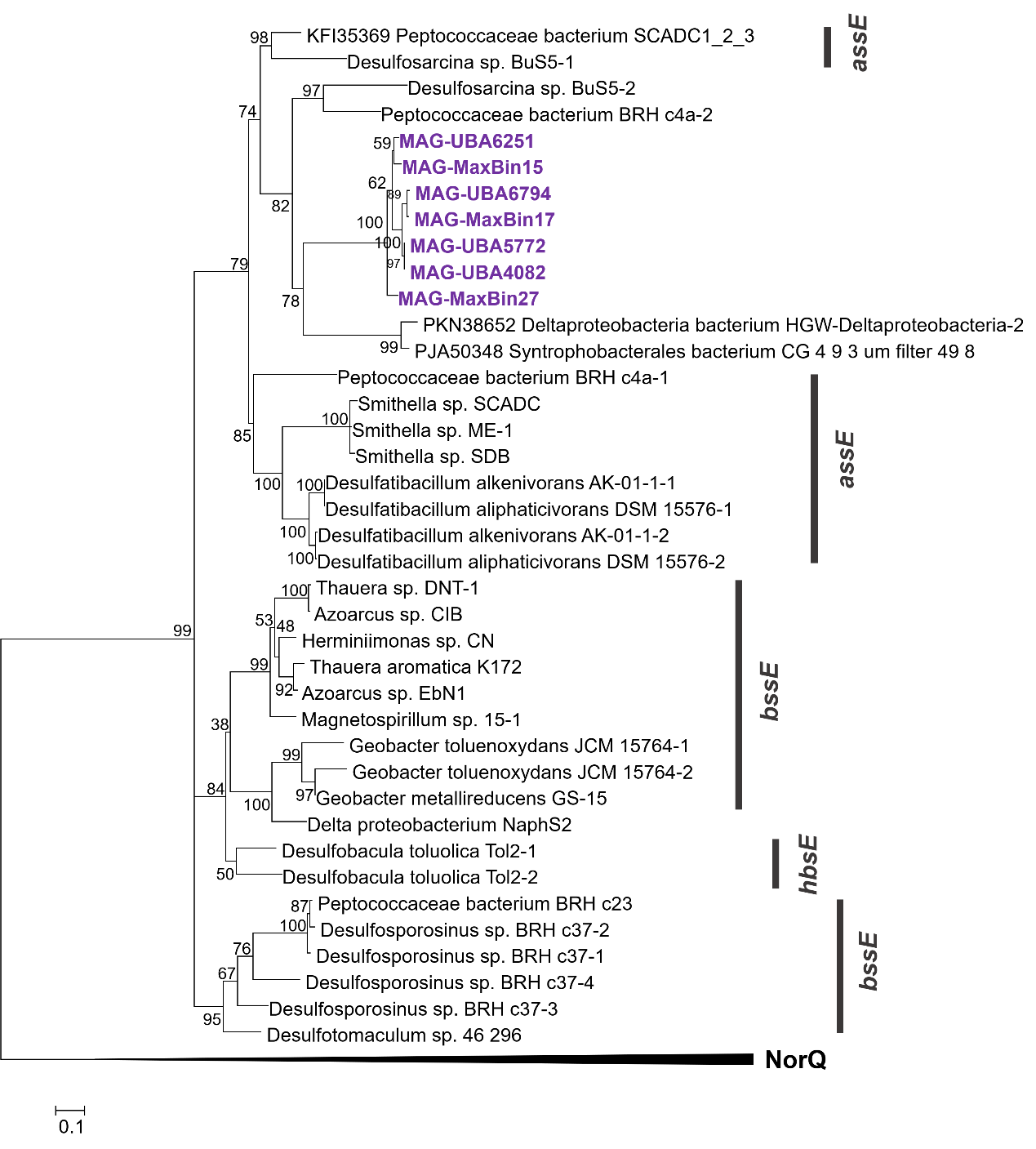


**Figure S7. Phylogenetic analysis of putative epsilon-subunit of fumarate-adding enzymes.** Sequences retrieved from Atribacterial MAGs were highlighted in purple. Maximum-likelihood tree were constructed in IQTREE Web Server with ‘standard’ model. Bootstrap values were determined using non-parametric bootstrapping with 1000 replicates and are shown on the tree.


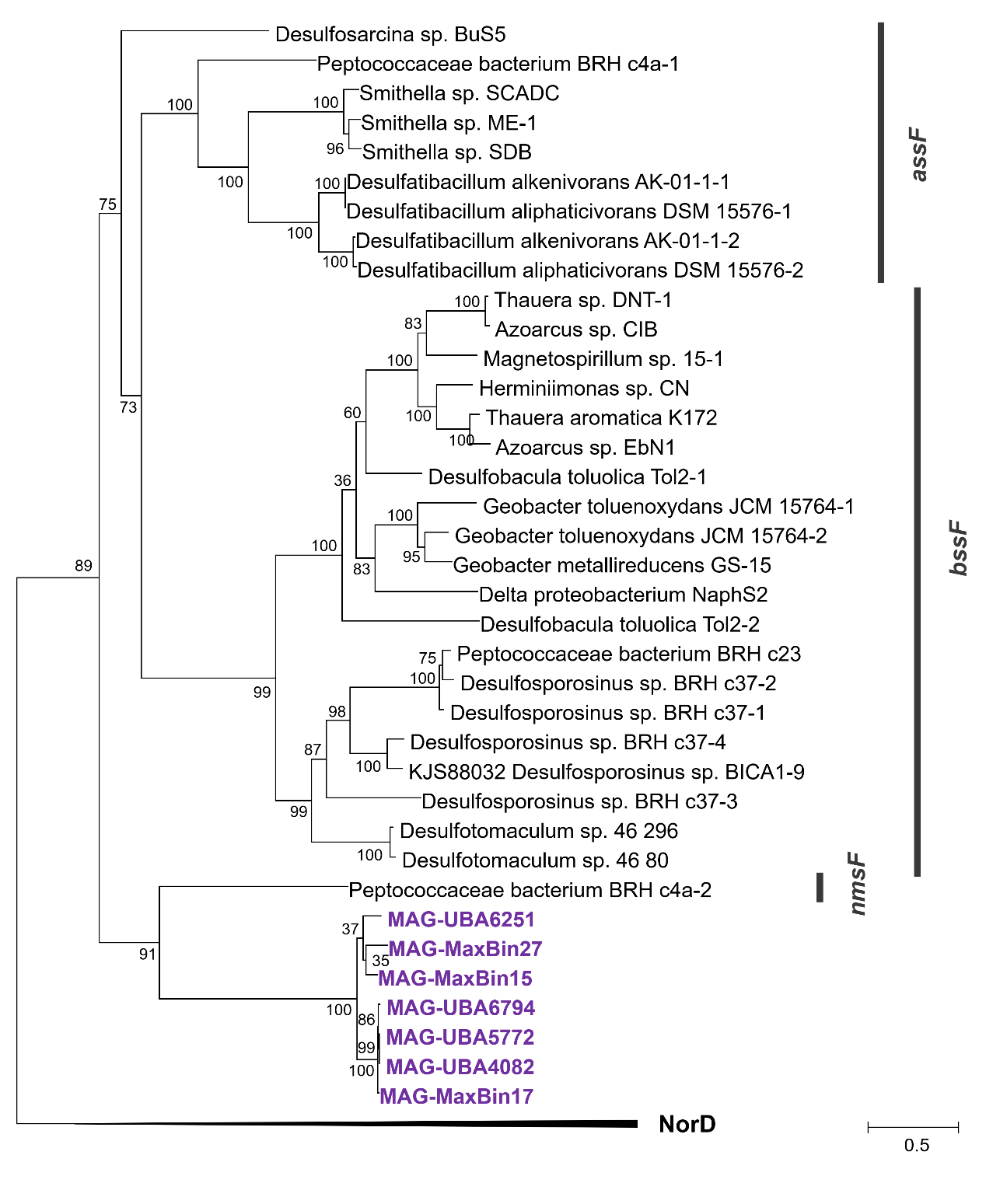


**Figure S8. Phylogenetic analysis of putative zeta-subunit of fumarate-adding enzymes.** Sequences retrieved from Atribacterial MAGs were highlighted in purple. Maximum-likelihood tree were constructed in IQTREE Web Server with ‘standard’ model. Bootstrap values were determined using non-parametric bootstrapping with 1000 replicates and are shown on the tree.


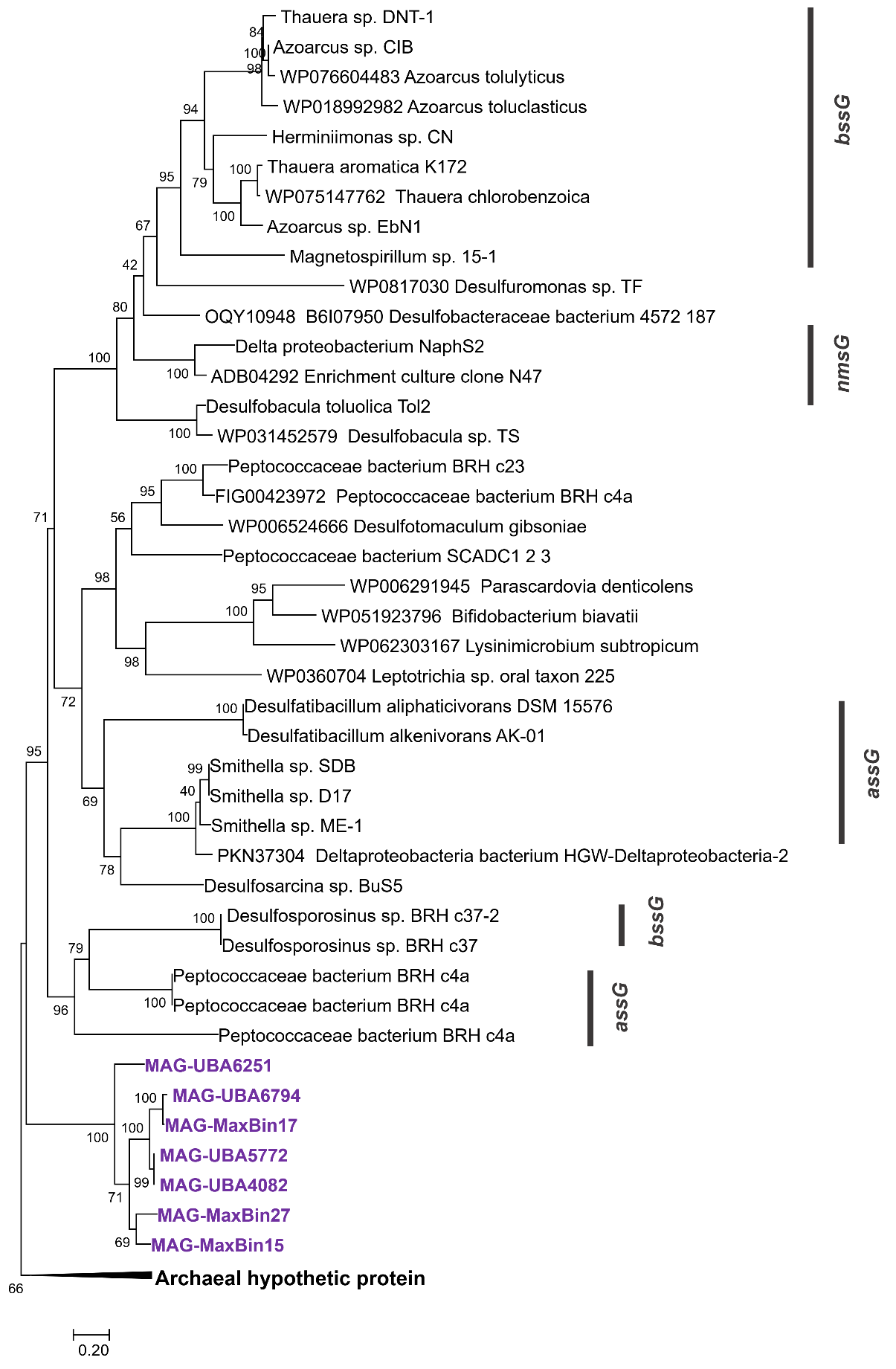


**Figure S9. Phylogenetic analysis of putative eta-subunit of fumarate-adding enzymes.** Sequences retrieved from Atribacterial MAGs were highlighted in purple. Maximum-likelihood tree were constructed in IQTREE Web Server with ‘standard’ model. Bootstrap values were determined using non-parametric bootstrapping with 1000 replicates and are shown on the tree.

**Table S1. Reference genomes used in this study (NCBI GeneBank, accessed on May 12, 2018).** The newmly assembled MAGs were marked in bold, and FAE-containing MAGs were marked in red.

| **GenBank / RAST Acc. No.** | **Genome Name** | **Completeness** | **Contamination** | **Strain heterogeneity** | **Reference** |
| --- | --- | --- | --- | --- | --- |
| AQRR01000001 | Atribacteria bacterium JGI 0000079-F20 | 0 | 0 | 0 | [10] |
| **DGDO01000089** | **Candidatus Atribacteria bacterium UBA3950** | 96.61 | 1.69 | 100 | [11] |
| APCU01000001 | Candidatus Caldatribacterium saccharofermentans OP9-77CS | 99.44 | 6.39 | 28.57 | [12] |
| APKF00000000 | Candidatus Caldatribacterium californiense OP9-cSCG | 96.61 | 0.15 | 0 | [12] |
| **6666666.334043** | **Maxbin015** | 98.31 | 0 | 0 | This study |
| ASLS01000001 | Atribacteria bacterium JGI 0000059-I14 | 16.95 | 0 | 0 | [10] |
| **6666666.334044** | **Maxbin017** | 96.61 | 0 | 0 | This study |
| **DKFO01000001** | **Candidatus Atribacteria bacterium UBA6794** | 78.81 | 0 | 0 | [11] |
| ASOZ01000001 | Atribacteria bacterium JGI 0000014-F07 | 8.33 | 0 | 0 | [10] |
| CDPL01000001 | Atribacteria bacterium JGI 0000014-B17 | 8.33 | 0 | 0 | [9][13] |
| ASOY01000001 | Atribacteria bacterium JGI 0000079-L04 | 35.34 | 0 | 0 | [10] |
| **DJVR01000031** | **Candidatus Atribacteria bacterium UBA6251** | 64.41 | 0 | 0 | [11] |
| **DIEO01000101** | **Candidatus Atribacteria bacterium UBA5772** | 94.54 | 4.49 | 0 | [11] |
| **DFYM01000072** | **Candidatus Atribacteria bacterium UBA4082** | 98.31 | 4.24 | 0 | [11] |
| **6666666.655183** | **Maxbin027** | 98.31 | 6.78 | 0 | This study |
| **PFIP01000081** | **Candidatus Atribacteria bacterium CG_4_8_14_3_um_filter_34_18** | 89.15 | 0 | 0 | [15] |
| **PEXH01000073** | **Candidatus Atribacteria bacterium CG08_land_8_20_14_0_20_33_29** | 84.62 | 2.56 | 0 | [15] |
| **MNYY01000032** | **Candidatus Atribacteria bacterium CG2_30_33_13** | 93.73 | 0.15 | 100 | [15] |
| **PFTV01000069** | **Candidatus Atribacteria bacterium CG_4_9_14_3_um_filter_33_16** | 89.2 | 1.28 | 0 | [15] |
| **PFFR01000114** | **Atribacteria bacterium CG17_big_fil_post_rev_8_21_14_2_50_34_11** | 91.37 | 0 | 0 | [15] |
| **PFKO01000153** | **Candidatus Atribacteria bacterium CG_4_10_14_3_um_filter_34_13** | 84.18 | 1.69 | 0 | [15] |
| AQRY01000001 | Atribacteria bacterium SCGC AB-164-G04 | 32.46 | 0 | 0 | [10] |
| **6666666.334062** | **Maxbin010** | 100 | 0.85 | 0 | This study |
| **MEYH01000001** | **Candidatus Atribacteria bacterium RBG_19FT_COMBO_35_14** | 93.59 | 3.85 | 0 | [16] |
| **NBMG01000116** | **Candidatus Atribacteria bacterium 4572_76** | 53.39 | 0 | 0 | [17] |
| CDPM01000001 | Atribacteria bacterium JGI 0000014-I22 | 22.41 | 0 | 0 | [9][13] |
| AQSW01000001 | Atribacteria bacterium SCGC AB-164-A22 | 31.03 | 0 | 0 | [10] |
| ASPA01000001 | Atribacteria bacterium SCGC AAA255-G05 | 62.16 | 3.99 | 80 | [10] |
| ASPC01000001 | Atribacteria bacterium SCGC AAA255-N14 | 51.28 | 0 | 0 | [10] |
| ASPB01000001 | Atribacteria bacterium SCGC AB-164-D21 | 32.2 | 0.85 | 100 | [10] |
| AQYX01000001 | Atribacteria bacterium SCGC AAA252-M02 | 58.73 | 2.97 | 60 | [10] |
| AWNT00000000 | Atribacteria bacterium JGI OTU-1 | 56.61 | 1.69 | 33.33 | [10] |
| ASZL01000001 | Atribacteria bacterium SCGC AB-164-M20 | 37.93 | 0 | 0 | [10] |
| ASOW01000001 | Atribacteria bacterium SCGC AB-164-L03 | 17.24 | 0 | 0 | [10] |
| ASLT01000001 | Atribacteria bacterium SCGC AAA255-E04 | 8.62 | 0 | 0 | [10] |

* This is predicted by CheckM to indicate contamination resulting from the presence of genomic fragments from multiple strains [18].

**Table S2. 107 marker genes used for initial clustering in Maxbin2 v2.2.3.**

**Table S3. Lineage specific tRNAs and marker genes used to evaluate genome completeness in CheckM v0.0.15.**

**Table S4. 400 proteins used to construct phylogenetic tree in PhyloPhlAn.**

**Table S5. Numbers of genes predicted to be involved in this study in all Atribacterial MAGs.**

**Table S6. Putative carbohydrate active enzymes (CAZY) conserved only in JS1 pan-genomes, OP9 pan-genomes or common to both.**

**File S1. Scaffolds file of Maxbin010**

**File S2. Scaffolds file of Maxbin015**

**File S3. Scaffolds file of Maxbin017**

**File S4. Scaffolds file of Maxbin027**

**References**

1. Huang Y, Gilna P, Li W. Identification of ribosomal RNA genes in metagenomic fragments. *Bioinformatics* 2009; **25**: 1338–1340.

2. Aziz RK, Bartels D, Best AA, DeJongh M, Disz T, Edwards RA, et al. The RAST Server: rapid annotations using subsystems technology. *BMC Genomics* 2008; **9**: 75.

3. Yamada KD, Tomii K, Katoh K. Application of the MAFFT sequence alignment program to large data - Reexamination of the usefulness of chained guide trees. *Bioinformatics* 2016; **32**: 3246–3251.

4. Talavera G, Castresana J. Improvement of phylogenies after removing divergent and ambiguously aligned blocks from protein sequence alignments. *Syst Biol* 2007; **56**: 564–577.

5. Kumar S, Stecher G, Tamura K. MEGA7: Molecular Evolutionary Genetics Analysis version 7.0 for bigger datasets. *Mol Biol Evol* 2016; **33**: msw054.

6. Yang J, Roy A, Zhang Y. Protein-ligand binding site recognition using complementary binding-specific substructure comparison and sequence profile alignment. *Bioinformatics* 2013; **29**: 2588–2595.

7. Zhang Y, Skolnick J. TM-align: A protein structure alignment algorithm based on the TM-score. *Nucleic Acids Res* 2005; **33**: 2302–2309.

8. Yarza P, Yilmaz P, Pruesse E, Glöckner FO, Ludwig W, Schleifer K-H, et al. Uniting the classification of cultured and uncultured bacteria and archaea using 16S rRNA gene sequences. *Nat Rev Microbiol* 2014; **12**: 635–645.

9. Nobu MK, Dodsworth J a, Murugapiran SK, Rinke C, Gies E a, Webster G, et al. Phylogeny and physiology of candidate phylum ‘Atribacteria’ (OP9/JS1) inferred from cultivation-independent genomics. *ISME J* 2016; **10**: 273–286.

10. Rinke C, Schwientek P, Sczyrba A, Ivanova NN, Anderson IJ, Cheng J-F, et al. Insights into the phylogeny and coding potential of microbial dark matter. *Nature* 2013; **499**: 431–437.

11. Parks DH, Rinke C, Chuvochina M, Chaumeil PA, Woodcroft BJ, Evans PN, et al. Recovery of nearly 8,000 metagenome-assembled genomes substantially expands the tree of life. *Nat Microbiol* 2017; **2**: 1533–1542.

12. Dodsworth JA, Blainey PC, Murugapiran SK, Swingley WD, Ross CA, Tringe SG, et al. Single-cell and metagenomic analyses indicate a fermentative and saccharolytic lifestyle for members of the OP9 lineage. *Nat Commun* 2013; **4**: 1854.

13. Lloyd KG, Schreiber L, Petersen DG, Kjeldsen KU, Lever MA, Steen AD, et al. Predominant archaea in marine sediments degrade detrital proteins. *Nature* 2013; **496**: 215–218.

14. Parks DH, Rinke C, Chuvochina M, Chaumeil P-A, Woodcroft BJ, Evans PN, et al. SupInfo-Recovery of nearly 8,000 metagenome-assembled genomes substantially expands the tree of life. *Nature Microbiology* . 2017.

15. Probst AJ, Castelle CJ, Singh A, Brown CT, Anantharaman K, Sharon I, et al. Genomic resolution of a cold subsurface aquifer community provides metabolic insights for novel microbes adapted to high CO2 concentrations. *Environ Microbiol* 2017; **19**: 459–474.

16. Anantharaman K, Brown CT, Hug LA, Sharon I, Castelle CJ, Probst AJ, et al. Thousands of microbial genomes shed light on interconnected biogeochemical processes in an aquifer system. *Nat Commun* 2016; **7**: 1–11.

17. Dombrowski N, Seitz KW, Teske AP, Baker BJ. Genomic insights into potential interdependencies in microbial hydrocarbon and nutrient cycling in hydrothermal sediments. *Microbiome* 2017; **5**: 106.

18. Parks DH, Imelfort M, Skennerton CT, Hugenholtz P, Tyson GW. CheckM : assessing the quality of microbial genomes recovered from isolates , single cells , and metagenomes. *Genome Res* 2015; **25**: 1043–1055.
