# Supplementary figures and images for: Anaerobic hydrocarbon degradation in candidate phylum ‘Atribacteria’ (JS1) inferred from genomics
Source: ISME J. 2019 Jun 6;13(9):2377–90. doi: 10.1038/s41396-019-0448-2 (PMC6776118; doi:10.1038/s41396-019-0448-2)

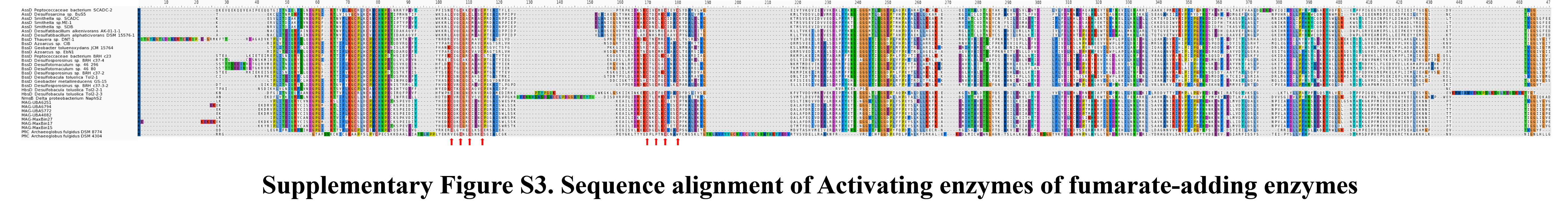

Supplement: Supplementary file 3 — Supplementary Figure S3 [file 41396_2019_448_MOESM3_ESM.png]
